# Spinal Cord Injury Significantly Alters the Properties of Reticulospinal Neurons: I. Biophysical Properties, Firing Patterns, Excitability, and Synaptic Inputs

**DOI:** 10.3390/cells10081921

**Published:** 2021-07-29

**Authors:** Ryan A. Hough, Timothee Pale, Jessica A. Benes, Andrew D. McClellan

**Affiliations:** 1 Division of Biological Sciences, University of Missouri, Columbia, MO 65211-6190, USA; rah021291@gmail.com (R.A.H.); timotheep@gmail.com (T.P.); jabenes01@aol.com (J.A.B.); 2 Interdisciplinary Neuroscience Program, University of Missouri, Columbia, MO 65211-6190, USA

**Keywords:** lamprey, axotomy, axonal regeneration, spinal cord injury, reticulospinal, biophysical properties

## Abstract

Following spinal cord injury (SCI) for larval lampreys, descending axons of reticulospinal (RS) neurons regenerate, and locomotor function gradually recovers. In the present study, the electrophysiological properties of uninjured (left)-injured (right) pairs of large, identified RS neurons were compared following rostral, right spinal cord hemi-transections (HTs). First, changes in firing patterns of injured RS neurons began in as little as 2–3 days following injury, these changes were maximal at ~2–3 weeks (wks), and by 12–16 wks normal firing patterns were restored for the majority of neurons. Second, at ~2–3 wks following spinal cord HTs, injured RS neurons displayed several significant changes in properties compared to uninjured neurons: (a) more hyperpolarized V_REST_; (b) longer membrane time constant and larger membrane capacitance; (c) increased voltage and current thresholds for action potentials (APs); (d) larger amplitudes and durations for APs; (e) higher slope for the repolarizing phase of APs; (f) virtual absence of some afterpotential components, including the slow afterhyperpolarization (sAHP); (g) altered, injury-type firing patterns; and (h) reduced average and peak firing (spiking) frequencies during applied depolarizing currents. These altered properties, referred to as the “injury phenotype”, reduced excitability and spiking frequencies of injured RS neurons compared to uninjured neurons. Third, artificially injecting a current to add a sAHP waveform following APs for injured neurons or removing the sAHP following APs for uninjured neurons did not convert these neurons to normal firing patterns or injury-type firing patterns, respectively. Fourth, trigeminal sensory-evoked synaptic responses recorded from uninjured and injured pairs of RS neurons were not significantly different. Following SCI, injured lamprey RS neurons displayed several dramatic changes in their biophysical properties that are expected to reduce calcium influx and provide supportive intracellular conditions for axonal regeneration.

## 1. Introduction

For all vertebrates, the pattern of rhythmic muscle burst activity during locomotor behavior is produced by central pattern generators (CPGs), which consist of neuronal oscillators that are distributed along the spinal cord and coupled by a coordinating system [1]. The spinal CPGs can produce the basic motor pattern for locomotion in the absence of sensory feedback [2]. Locomotion is initiated, maintained, and regulated by a brain command system, the output of which consists of reticulospinal (RS) neurons whose descending axons activate spinal CPGs [1,3]. Above a threshold level of activity for RS neurons, further increases in the activity of these neurons is correlated with an increase in the frequency of rhythmic spinal locomotor activity [4]. In addition, increases in the intensity of experimental stimulation in medullary reticular nuclei increase the frequency of spinal locomotor activity and locomotor movements [5,6,7].

After a complete upper spinal cord injury (SCI), descending brain-spinal pathways, including projections of RS neurons, are disrupted, as are ascending spinal-brain pathways. The disruption of descending inputs of RS neurons to spinal CPGs results in loss of locomotor activity, as well as other motor functions, below the lesion site [8]. Following complete SCI in “higher” vertebrates, including birds and mammals, the injured descending axons of RS neurons normally do not regenerate, resulting in permanent paralysis [9,10]. The lack of axonal regeneration and functional recovery is due to several mechanisms, including inhibitory factors for axonal outgrowth in the injured CNS as well as secondary injury effects [9,10,11,12]. In contrast, following complete SCI in “lower” vertebrates, including lampreys, fish, and some amphibians, the axons of injured RS neurons regenerate and reconnect with spinal CPGs below the lesion site, resulting in recovery of locomotor and other motor functions within a few weeks (reviewed in [13,14,15]). Thus, the CNS of lower vertebrates is a permissive environment for axonal regeneration, and secondary injury effects appear to be minimal [15].

Following complete upper SCI, lampreys are paralyzed below the injury site, as would be the case for any vertebrate. However, at ~2 week (wk) recovery times, animals begin to generate weak locomotor-like muscle burst activity just below the lesion site, and by 8 weeks (wks), animals have almost completely recovered and display virtually normal locomotor movements and muscle burst activity [16] During this remarkable recovery process, the descending axons of RS neurons extend through the transection site, regenerate for progressively greater distances in the spinal cord with increasing recovery times [17,18,19], and reconnect with spinal CPGs [16,20]. However, even at relatively long recovery times (e.g., 32 wks), the restored projections of RS neurons to CPGs in the middle and lower spinal cord are significantly less than the projections of these neurons in normal animals [17,18]. Thus, incomplete axonal regeneration of RS neuron projections can support virtually complete locomotor recovery because of several compensatory mechanisms that make up for this incomplete regeneration and facilitate full recovery (reviewed [14,15]).

**Figure 1:**
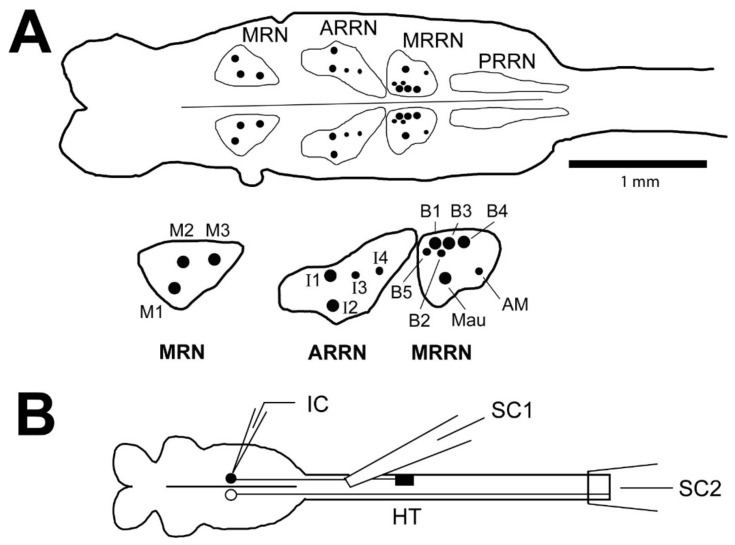
(**A**) (upper) Tracing of a larval lamprey brain (left) and rostral spinal cord (right) showing outlines around reticular nuclei: MRN=mesencephalic reticular nucleus; ARRN (anterior), MRRN (middle), and PRRN (posterior) rhombencephalic reticular nuclei. (lower) Enlargement of left reticular nuclei that contain large, uniquely identified reticulospinal (RS) neurons (Müller cells) that have *ipsilateral* projecting descending axons: M cells (M1-M3) in the MRN; I cells (I1-I4) in the ARRN; and B cells (B1-B5) in the MRRN. Mauthner (Mau) and auxiliary Mauthner (AM) cells are other identified RS neurons located in the MRRN that have *contralateral* projecting descending axons. In addition, all reticular nuclei (MRN, ARRN, MRRN, PRRN) contain numerous smaller, unidentified RS neurons that are omitted for simplicity. (**B**) Diagram of isolated brain-spinal cord preparation (not to scale), showing the brain (left) and rostral spinal cord (right), right spinal cord hemi-transection (HT) at 10% body length (BL, relative distance from the anterior tip of the head), and intracellular micropipette (IC) for recording from RS neurons. Spinal cord suction electrodes (SC1, SC2) were used to record RS neuron-evoked orthodromic action potentials rostral and caudal to spinal HTs to determine if a given neuron was injured (●) or uninjured (○) (see Methods).

For larval lamprey, small, unidentified RS neurons are both necessary and sufficient for initiation of locomotion [21,22]. Although large, identified lamprey RS neurons (Müller cells: M cells; I cells; and B cells; see Figure 1A), which are the focus of the present study, are not necessary for basic locomotion [23], these neurons probably do contribute to certain aspects of locomotor function. First, these neurons rhythmically burst during locomotor activity [24] and during locomotor movements [25,26]. Second, higher order brain locomotor centers project directly or indirectly to these large RS neurons [21,27]. Importantly for the present study, Müller cells and small, unidentified RS neurons display similar changes in biophysical properties in response to SCI [28].

Large, identified lamprey RS neurons (Müller cells) have *ipsilateral* descending axons [23,29]. Thus, following right upper spinal cord hemi-transections (HTs), large, identified right RS neurons are injured while left neurons remain uninjured [28]. At relatively short recovery times (2-3 wks) following these spinal cord HTs, virtually all uninjured (left) “B” cells (B1, B3, B4; see Figure 1A) displayed smooth, continuous repetitive firing in response to depolarizing current pulses [28]. In addition, action potentials (APs) of uninjured RS neurons usually were followed by three sequential afterpotential components: fast afterhyperpolarization (fAHP); afterdepolarization (ADP); and slow AHP (sAHP). In contrast, most injured (right) “B” cells displayed injury-type repetitive firing patterns: single, short burst/single AP; or short multiple bursts. In addition, the ADP and sAHP afterpotential components for these neurons were virtually absent. At relatively long recovery times (12–16 wks), most right “B” cells fired smooth trains of APs [28], and ADP and sAHP afterpotential components were restored. It is likely that the restoration of normal firing patterns and other properties for injured RS neurons is due, in part, to these neurons forming synapses below the injury site to restore target-derived neurotrophic support [30]. Finally, following right spinal HTs, in right reticular nuclei, which contained mostly injured RS neurons, mRNA levels for high-voltage activated (HVA) calcium and calcium-activated potassium (SK) channels, both of which contribute to the sAHP, were significantly reduced at short recovery times and were restored at long recovery times [28]. We hypothesize that a decrease in expression of calcium channels for injured RS neurons is one of the mechanisms that provides a supportive intracellular environment for axonal regeneration ([28]; reviewed in [15]).

The purpose of the present study was to extend the analysis following spinal HTs to include additional large, identified injured lamprey RS neurons (M cells, I cells, B cells; see Figure 1A) and additional recovery times (2–3 days to 12–16 wks) as well as to test possible mechanisms for the differences in properties of uninjured and injured RS neurons. Many studies have described the effects of axotomy on the biophysical properties of neurons, but the effects often are quite variable and difficult to generalize (see Discussion). In the present study, in addition to characterizing the effects of SCI (axotomy) on the biophysical properties of injured RS neurons, we are particularly interested in linking these altered properties to the ability of these neurons to regenerate their axons. For most experiments, recordings were made from uninjured (left)-injured (right) pairs of large lamprey RS neurons following right spinal cord HTs. This approach tended to normalize for variations in properties of different RS neurons and, unlike previous studies [28,30], often allowed comparisons of uninjured-injured pairs of neurons within the same animal. For injured RS neurons, the time course for altered firing patterns was established, and the biophysical properties, firing patterns, excitability, and sensory-evoked synaptic responses were compared for pairs of uninjured and injured RS neurons. Notably, following SCI, injured lamprey RS neurons displayed several dramatic changes in their biophysical properties that are expected to reduce calcium influx and provide supportive intracellular conditions for axonal regeneration. Because lampreys exhibit robust axonal regeneration and impressive behavioral recovery following SCI, a better understanding of the neuronal changes that occur for RS neurons following injury is important for determining the cellular and molecular conditions that support successful axonal outgrowth for these neurons. This and other information might provide insights for developing methods to enhance axonal regeneration following SCI in higher vertebrates, including perhaps humans.

## 2. Materials and Methods

### 2.1. Animal Care

Larval sea lampreys (*Petromyzon marinus*) (Length, L = 80–147 mm), which were collected from streams and rivers in Michigan or Massachusetts, were used for all experiments and were maintained in ~10 L aquaria at 22–24 °C. For all surgical procedures, animals were anesthetized in ~200 mg/L tricaine methanosulphonate (MS-222; Crescent Research Chemicals; Phoenix, AZ, USA). The procedures in this study have been approved by the Animal Care and Use Committee (ACUC) at the University of Missouri USA (Protocol 9410, 1 August 2020).

### 2.2. Neurophysiological Properties of Uninjured and Injured RS Neurons

#### 2.2.1. Animal Groups

For the present study, several animal groups were used (see Results for n values): (a) normal animals without spinal lesions; (b) experimental animals with right spinal cord hemi-transections (HTs) at 10% body length (BL, normalized distance from the anterior head); and (c) experimental animals in which the lateral spinal tracts (i.e., left and right lateral one third of the spinal cord) and dorsal columns (i.e., dorsomedial spinal cord) were lesioned at 10% BL. For performing spinal lesions, a ~5 mm dorsal incision was made at 10% BL, the spinal cord was exposed, and lesions were made with iridectomy scissors and fine forceps. Subsequently, the incisions were manually closed, and animals were returned to their aquariums to recover, usually for 2–3 weeks (wks) but in some cases for 2–3 days to 16 wks. In the present study, intracellular recordings were made from large, identified RS neurons (Figure 1A; Müller cells; see [18]), which have *ipsilateral*, descending axons [23,29]. Thus, right spinal cord HTs injured most right RS neurons while leaving most left neurons uninjured. This approach tended to normalize for possible variations in properties of different RS neurons, and, in contrast to previous studies [28,30], often allowed comparisons of uninjured (left)-injured (right) pairs of RS neurons within the same animal (see Methods below).

#### 2.2.2. Isolated Brain-Spinal Cord Preparation

Animals were anesthetized and fully transected just below the most caudal gill. A ventral approach was used to expose the brain and rostral spinal cord, as previously described [28,30]. The brain and rostral spinal cord were removed and pinned dorsal-side-up on a small rectangular strip of Sylgard (Corning Co; Midland, MI, USA), and transferred to a recording chamber containing cold (~6–8 °C) oxygenated, lamprey Ringer’s solution: 10 mM HEPES; 130 mM NaCl; 2.1 mM KCl; 2.6 mM; CaCl_2_; 1.8 mM MgCl_2_; and 4.0 mM Dextrose (pH = 7.4). For some experiments, 0.5 mM kynurenic acid (KYNA; Sigma Chemical Co., St. Louis, MO, USA) was added to the bath to reduce spontaneous electrical activity [31]. No obvious differences in neuron properties were observed with or without KYNA.

Suction electrodes were placed on the right, dorsal surface of the spinal cord above the spinal HTs (~8–9% BL; see SC1 in Figure 1B) and around the caudal spinal cord below the HTs (~13–17% BL; see SC2 in Figure 1B). Thus, during stimulation of action potentials in RS neurons (see below), the presence or absence of orthodromic responses caudal to the spinal cord HTs was used to determine if a particular neuron was uninjured or injured, respectively (e.g., see Figure 5(A2,B2)).

Intracellular recordings were performed from large, identified RS neurons (Müller cells: usually M2, M3, I1, B1, B3, B4; see Figure 1A) with sharp-tipped micropipettes pulled from thin-wall glass tubing (WPI, Sarasota, FL, USA) and filled with 5 M potassium acetate (R_e_ ~50–70 MΩ). Micropipettes were inserted into a holder that was plugged into the head stage of an intracellular amplifier (Axoclamp 2-A; Axon Instruments, Foster City, CA, USA), and the head stage was mounted on a motorized manipulator that could rapidly advance the tip of the micropipette in the z-axis in ~1 μm increments.

#### 2.2.3. Passive Electrical Properties

First, the resting membrane potential (V_REST_) was measured. Second, with the discontinuous current clamp mode (DCC; f_s_ ~4–6 kHz), small, hyperpolarizing current pulses (ΔI_m_ = 0.01–3.0 nA, 0.2 s) were applied to elicit relatively small membrane potential hyperpolarizations (ΔV_m_ < 1–5 mV) and to measure passive electrical properties of uninjured and injured RS neurons (see Table 3 for n values). Multiple voltage traces were averaged (usually ≥10 traces), and the membrane input time constant (τ_in_) was determined as the time for ΔV_m_ to reach 63% of maximum. Membrane input resistance (R_in_ = ΔV_m_/ΔI_m_) and membrane input time constant (τ_in_) were determine and used to calculate membrane input capacitance (C_in_ = τ_in_/R_in_).

#### 2.2.4. Action Potential Properties

With the continuous current clamp (“bridge”) mode, single action potentials (APs) were elicited for uninjured and injured RS neurons by applying relatively short duration depolarizing current pulses (0.1–10 ms, +10 nA). The APs were elicited after termination of the current pulses when applied current was no longer passing through the micropipette resistance. The following biophysical properties were measured (see Table 3 for n values): (a) V_AP_—the amplitude from V_REST_ to the peak of the AP; (b) D_AP_—the duration of the AP at half maximal amplitude; and dV_m_/dt_rise_ and dV_m_/dt_fall_—the maximum slope of the rising and falling phases of action potentials, respectively. The dV_m_/dt rise and fall values were determined by differentiating the first 80 ms of APs and constructing phase plane plots for which −dV_m_/dt was plotted against V_m_.

#### 2.2.5. Afterpotentials

Immediately following the repolarizing phase of APs, uninjured RS neurons often displayed three sequential afterpotentials [28]: (a) fAHP—fast afterhyperpolarization; (b) ADP—afterdepolarization; and (c) sAHP—slow AHP. Because of the relatively small amplitudes of these afterpotentials, several AP sweeps were averaged, and the following parameters were measured (see Table 3 for n values): (a) V—amplitude of a given afterpotential component relative to V_REST_; (b) D—half-amplitude duration for each afterpotential component; and (c) d—delay from the peak of an AP to the peak of a given afterpotential component. If one of the afterpotential components was clearly absent, the amplitude of that component was assigned a value of 0.0, and “D” and “d” for that component were left blank (i.e., not measurable). If an afterpotential component appeared to be present but was not measurable (e.g., peak of apparent fAHP or ADP was depolarized or hyperpolarized, respectively, relative to V_REST_), then all of the parameters for that component were left blank (i.e., not measurable).

#### 2.2.6. Repetitive Firing Patterns

Threshold voltage (ΔV_TH_), threshold current (I_TH_), and repetitive firing patterns for uninjured and injured RS neurons were determined by applying depolarizing current pulses (0.1–10 nA, 2.0 s) using the DCC mode (f_s_ ~4–6 kHz). The instantaneous AP firing (spiking) frequency (Freq) was determined by a custom electronic device, as previously described [28,30,32]. When uninjured RS neurons were depolarized to V_TH_, they often responded with one or very few APs, and as such, V_TH_ was measured as the steady state membrane potential following an AP, and ΔV_TH_ = V_TH_ − V_REST_. In contrast, injured RS neurons that were depolarized to V_TH_ often responded with a single AP or short burst of APs. However, at the onset of a depolarizing current pulse just below V_TH_, injured RS neurons usually displayed a transient depolarization (Hough and McClellan, in preparation), which was obscured at greater depolarizations when APs were present. Therefore, during application of depolarizing current pulses to injured RS neurons, V_TH_ was measured as the peak of the transient depolarization that was just sub-threshold for eliciting APs, and again ΔV_TH_ = V_TH_ − V_REST_.

Repetitive firing patterns of RS neurons were characterized at *intermediate depolarizing current levels* (see rationale in Section 3.3 of Results) based on four possible outcomes: (a) smooth train of APs—spikes were elicited during the entire current pulse and with a smooth, progressive decrease in the instantaneous firing frequency due to spike frequency adaptation (SFA; [32]); (b) irregular firing—continuous firing during the entire current pulse, but with multiple variations in instantaneous spiking frequency of ≥20%; (c) multiple bursts—several relatively short, consecutive bursts of APs; and (d) relatively short burst or single AP—a relatively brief burst of APs or one AP at the beginning of depolarizing current pulses. In addition to application of current pulses, sinewave currents (F = 1.0, 2.0, 3.0 Hz) were applied to some RS neurons. Compared to applied current pulses, application of sinusoidal current waveforms more closely mimicked the rhythmic oscillations of membrane potential (V_m_) that occur for these neurons during locomotor activity [24].

The average and peak firing (spiking) frequencies were determined for uninjured (left) and injured (right) pairs of RS neurons. For application of depolarizing current pulses, the average spiking frequency for a given value of current injection was calculated as the number of APs divided by 2.0 s. For applied sinusoidal current injection, average spiking frequency was equal to the number of APs per cycle times 2 * F, where F was the frequency of the sinewave. For both applied current pulses and sinusoidal current waveforms, the minimum interval between consecutive APs (Δt) was used to calculate peak spiking frequencies (=1/Δt). These data were used to construct frequency-current (F-I) plots (see Figure 7).

### 2.3. Manipulations of the sAHP

The sAHP, which is involved in spike-frequency regulation [33,34], usually is present for uninjured RS neurons but is largely absent for injured RS neurons [28]. To examine the effects of the sAHP on repetitive firing, the sAHP was removed after each AP for uninjured RS neurons, and for injured neurons an sAHP was introduced after each AP. Ideally, this procedure should be performed using dynamic clamp [35], but it is very difficult to effectively voltage clamp these large RS neurons over the full V_m_ range because of their extensive dendritic trees [36]. Therefore, the sAHP was removed or introduced using an injected current waveform to mimic the sAHP. Specifically, each AP triggered a delayed pulse (0–50 ms) that was applied to a resistor-capacitor circuit, which had adjustable charging and discharging time constants (0–100 ms and 0–200 ms, respectively), and the amplitude and polarity of the output waveform could be adjusted to mimic the shape of the sAHP. First, for uninjured RS neurons, single APs were triggered in the DCC mode, and following each AP, a *positive* current waveform was injected to remove the neural sAHP waveform (see Figure 6(A1,A3)). Subsequently, 2.0 s depolarizing current pulses were applied to elicit repetitive firing, and after each AP, the neural sAHP was removed. Second, for injured RS neurons, a *negative* current waveform was injected following each AP to introduce a typical sAHP waveform (−1 to −3 mV amplitude; ~100 ms half-amplitude duration; see Figure 6(B1,B3)), either following single APs or following each AP during repetitive firing.

### 2.4. Data Acquisition/Storage and Statistics

All electrophysiological data were stored on tape (Neurodata DR890; Cygnus Technologies; Delaware Water Gap, PA, USA; 11 kHz sampling rate per channel) as well as acquired by a custom data acquisition and analysis system (DT3016 data acquisition board; Data Translation, Marlboro, MA, USA). Biophysical properties are presented as mean ± standard deviation (SD). First, for animals that had recovered for 2–3 wks following right spinal cord HTs at 10% BL, passive properties, action potential characteristics, and afterpotential features for uninjured (left)-injured (right) pairs of RS neurons (n = 38 pairs of neurons; N = 27 animals) were compared. Not all properties or features were measured or could be measured for every RS neuron (see above). For passive properties and action potential properties, n values for uninjured and injured neurons were relatively large and within 0–2 of each other. Therefore, we omitted the few unpaired data points so that all the data points for these parameters were paired, n values were equal for uninjured and injured neurons (see Table 3), and the data were analyzed with paired *t*-tests (InStat, La Jolla, CA, USA). For afterpotential parameters, n values for uninjured and injured RS neurons were quite different (see Table 3) (note: V_sAHP_ only had 31 paired data points out of 35 maximum points). Also, for injured RS neurons, the “D” and “d” parameters for the ADP and sAHP (whose amplitudes often = 0.0) often could not be measured and had low n values. Because many afterpotential data points would need to be omitted, thereby reducing statistical power, to allow for paired *t*-tests, to be consistent, all afterpotential data were analyzed with unpaired *t*-tests (see Table 3). However, omitting unpaired data points for V_fAHP_, D_fAHP_, d_fAHP_, V_ADP_, or V_sAHP_ and conducting paired *t*-tests on the remaining data did not change the statistical results for any of these particular parameters.

Second, for animals that had recovered for 2–3 wks following right spinal cord HTs at 10% BL, repetitive firing properties (F-I plots) for uninjured (left)-injured (right) pairs of RS neurons (n = 43 pairs of neurons; N = 33 animals) were compared using a Sign test (https://www.graphpad.com/quickcalcs/binomial1.cfm; one-tailed *p* value; accessed on 19 July 2021), which calculated the *p* value based on the fraction of pairs for which spiking frequencies for uninjured neurons were clearly higher than those for injured neurons (e.g., see Figure 7). For all statistics, significance was assumed for *p* ≤ 0.05. The n values and N values for additional neurophysiological experiments using isolated brain-spinal cord preparations are listed in the Results.

### 2.5. Sensory-Evoked Synaptic Responses of Uninjured and Injured RS Neurons

For normal animals, in vitro brain/spinal cord preparations were set up (see Figure 8A), as previously described [37,38] and placed in a recording chamber containing lamprey Ringer’s solution (~6–8 °C), and 20 mg/L D-tubocurarine (Sigma) was added to block possible contractions of remaining musculature. For experimental animals, right spinal cord HTs were performed at 10% BL, as described above, and following a 2–3 wk recovery time, in vitro brain-spinal cord preparations were set up (see Figure 9A). Suction electrodes were placed above and below the spinal cord HTs to determine the injury status of RS neurons, as described above. Using conventional current clamp (“bridge”) mode, intracellular recordings were made from RS neurons in normal animals and from uninjured (left)-injured (right) pairs of RS neurons in animals with spinal cord HTs. Oral hood stimulating electrodes each consisting of pairs of copper wires, insulated except at the tips (0.41 mm diameter wires; tip separation ~1.5 mm), were placed symmetrically in contact with the right and left lateral parts of the oral hood (see Figures 8A and 9A). For each RS neuron, the oral hood was stimulated on the right side and then on the left side via stimulus isolation units with short current pulses (1.0 ms, 0.0–2.0 mA at ~0.25 Hz) to activate trigeminal sensory neurons and elicit sensory-evoked synaptic responses in RS neurons [37,38]. Lastly, small hyperpolarizing current pulses were applied to uninjured-injured pairs of RS neurons to determine membrane input resistance (R_in_), as described above.

Sensory-evoked synaptic responses in RS neurons were variable and consisted of excitatory postsynaptic potentials (EPSPs), mixed PSP-EPSPs, and inhibitory postsynaptic potentials (IPSPs). However, for mixed PSP-EPSPs, the major component of the synaptic response was always the EPSP, and only one purely IPSP synaptic response was observed for a single uninjured neuron. Therefore, the following analyses were performed on the amplitudes of EPSPs and the EPSP component of mixed PSP-EPSPs, both of which will be referred to as “EPSPs”. First, for normal animals, evoked EPSPs were recorded from each RS neuron (n = 20 neurons; N = 9 animals) in response to contralateral and then ipsilateral stimulation of the oral hood based on stimulus thresholds. Specifically, EPSPs were recorded in response to the minimum oral hood-stimulating current for eliciting a synaptic potential (1 T = threshold stimulating current), as well at 1.5 T and 2 T. This experimental paradigm compensated for slight differences in the effectiveness of the left and right stimulating electrodes and variations in sensory receptor thresholds. For each stimulus intensity (1 T, 1.5 T, 2 T), sweeps of synaptic responses were averaged (usually ≥10 sweeps) for each RS neuron (see Figure 8B). Because EPSP amplitudes could be quite variable, outlier averaged EPSP amplitudes were omitted using Grubb’s test (http://contchart.com/outliers.aspx; accessed on 19 July 2021). In addition, for 1.5 T or 2 T stimulus intensities, APs sometimes were elicited such that EPSP amplitudes could not be measured, making pairwise comparisons sometimes problematic. Because n values for contralateral and ipsilateral stimulation usually were not equal, to be consistent, for each stimulus intensity, contralaterally-evoked EPSP amplitudes were compared to ipsilaterally-evoked EPSP amplitudes using an unpaired *t*-test, with Welch correction when appropriate (InStat) (see Figure 8C). However, omitting unpaired data points and conducting paired *t*-tests on the remaining data did not change the statistical results. Significance was assumed for *p* ≤ 0.05.

Second for experimental animals that had recovered for 2–3 wks following right spinal cord HTs at 10% BL, the contralateral oral hood was stimulated at 1 T, 1.5 T, and 2 T for uninjured (left) and injured (right) pairs of RS neurons (n = 11 left-right pairs; N = 10 animals) (see Figure 9). Outlier EPSP values were omitted using Grubb’s test. Because n values for uninjured and injured RS neurons usually were not equal, to be consistent, for each stimulus intensity, the contralaterally-evoked EPSP amplitudes were compared for injured and uninjured pairs of RS neurons using an unpaired *t*-test, with Welch correction when appropriate (InStat) (see Figure 10A). A similar analysis was used to compared ipsilaterally-evoked EPSPs for injured and uninjured pairs of RS neurons (see Figure 10B). However, omitting unpaired data points and conducting paired *t*-tests on the remaining data did not change the statistical results. Significance was assumed for *p* ≤ 0.05.

## 3. Results

### 3.1. Time Course of Altered Firing Patterns for Injured RS Neurons Following SCI

**Figure 2:**
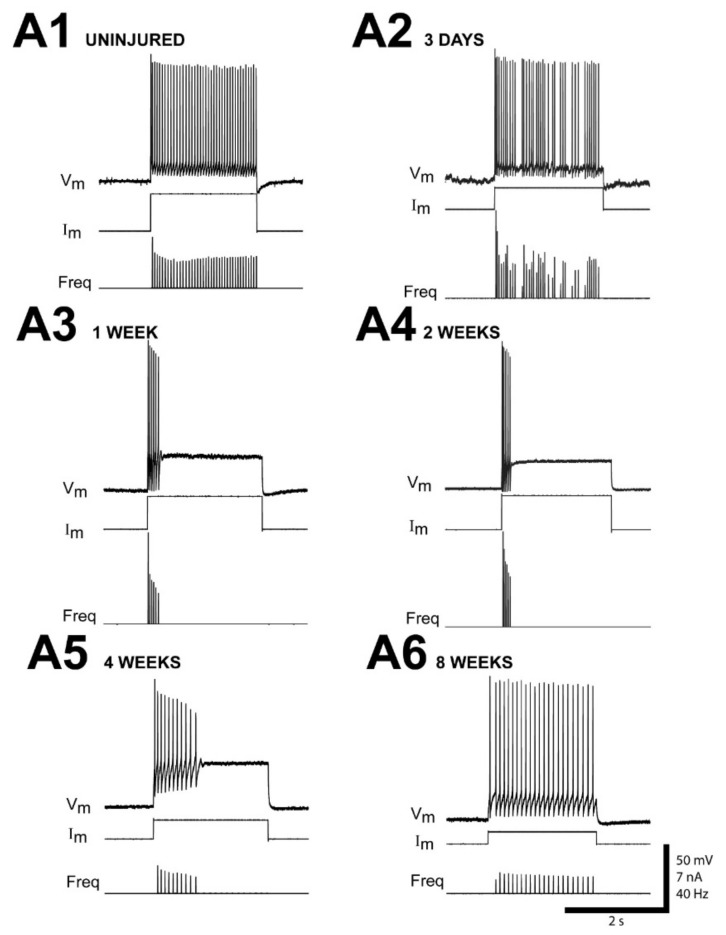
Examples of time course of altered firing patterns for RS neurons following right spinal cord HTs at 10% BL (see Figure 1B). Membrane potential (V_m_), injected current (I_m_), and instantaneous firing frequency (Freq). (**A1**) Smooth continuous repetitive firing of an uninjured (left) “B4” large, identified RS neuron (Müller cell, see Figure 1A,B; 2 wk recovery time) in response to a 2.0 s depolarizing current pulse. (**A2**–**A6**) Repetitive firing patterns of injured (right) “B4” RS neurons from different animals at various recovery times in response to 2.0 s depolarizing current pulses.

In our previous study, the firing patterns of uninjured and injured “B” cells (see Figure 1A) were determined at 2–3 wks and 12–16 wks following right spinal cord HTs at 10% BL [28]. In the present study, the firing patterns of additional injured RS neurons (M1-M3, I1-I4, B1-B5; see Figure 1A) were determined for additional recovery times from 2–3 days to 12–16 wks (Figure 2, Table 1).

**Figure 3:**
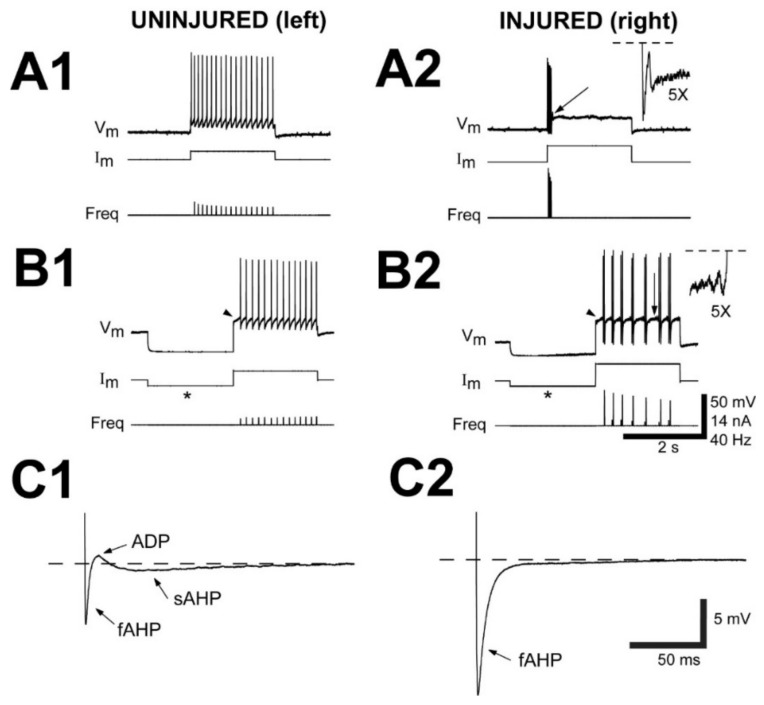
Recordings from uninjured (left)-injured (right) pairs of RS neurons from animals that had recovered for 2-3 wks following right spinal cord HTs at 10% BL (see Figure 1B). (**A1**) Left uninjured “B4” and (**B1**) left uninjured “M2” RS neurons (see Figure 1A) in the same brain fired a smooth, continuous train of action potentials (APs) during a 2.0 s depolarizing current pulse. (**A2**) Right injured “B4” and (**B2**) right injured “M2” RS neurons (paired with left uninjured neurons in **A1** and **B1**) fired a single burst or multiple short bursts, respectively, in response to depolarization. Membrane potential (V_m_), injected current (I_m_), and instantaneous firing (spiking) frequency (Freq). Note “delayed excitation” (DE, arrowheads) for both (**B1**) uninjured and (**B2**) injured “M2” neurons following a hyperpolarizing current prepulse (*). Insets (5× enlargement) and arrows (**A2,B2**) indicate V_m_ “resonance”, which was manifested as (**A2**) decaying V_m_ oscillations following a single burst or (**B2**) growing V_m_ oscillations between multiple bursts (dashed lines indicate truncated APs; fAHP is truncated in A2). (**C**) Afterpotentials (arrows) following the repolarizing phases of action potentials for a (**C1**) left uninjured “B3” neuron and (**C2**) right injured “B3” neuron in the same brain: fast afterhyperpolarization (fAHP); after depolarization (ADP); and slow AHP (sAHP). Dashed horizontal lines indicate V_REST_.

At all recovery times following right spinal cord HTs at 10% BL, most uninjured (left) RS neurons fired a smooth, continuous train of APs that occurred during the entire above-threshold 2.0 s depolarizing current pulse (Figure 2(A1) and Figure 3(A1,B1); see later in Results for further details). At 2–3 day recovery times, ~70% of injured (right) RS neurons (n = 9/13 neurons) exhibited irregular repetitive firing patterns in response to depolarizing current pulses (Figure 2(A2)), while 23% of the neurons fired smoothly (Table 1). At 1 wk recovery times, injured RS neurons began to display clear injury-type firing patterns (Table 1, Figure 2(A3)). At 2–3 wk recovery times, 76% of injured RS neurons (n = 110/145) displayed injury-type firing patterns: single, short burst of APs/single AP; or multiple short bursts of APs (Table 1, Figure 2(A4) and Figure 3(A2,B2)). At 4–8 wk recovery times, there was an increase in the percentage of RS neurons displaying smooth repetitive firing and a decrease in the percentage exhibiting injury-type firing patterns (Table 1, Figure 2(A5,A6)). At 12–16 wk recovery times, almost 70% of right RS neurons (n = 13/19) displayed smooth firing. However, at these relatively long recovery times, ~80% of B1, B3, and B4 cells (n = 11/14 neurons) had recovered normal, smooth firing patterns, while only ~40% of M2, M3, and I1 cells (n = 2/5 of neurons) had recovered normal firing patterns. In addition, except for M3, which is the poorest axonal regenerator among Müller cells [18] and accounted for only ~5% of neurons sampled at 12–16 wk recovery times, the percentages of the other different RS neurons that were recorded from at different recovery times were roughly comparable (Table A1 in Appendix A; see Discussion). In conclusion, one the characteristics of injured RS neurons, injury-type firing patterns, appears to be most pronounced at ~2–3 wks following SCI.

At 2–3 wk recovery times following right spinal cord HTs, the different large, identified injured (right) RS neurons (M2, M3, I1, B1, B3, B4; see Figure 1A) displayed relatively similar distributions of injury-type firing patterns (Table 2; n = 142 neurons, N = 53 animals). Typically, in response to 2.0 s depolarizing current pulses, many of the different RS neurons exhibited a single short burst/single AP (~35–60%), while fewer neurons displayed multiple short bursts (~25–35%), and still fewer neurons exhibited irregular firing (~0–20%). Thus, at 2–3 wk recovery times, different large, identified injured RS neurons did not appear to have distinct responses to SCI, at least with regard to repetitive firing patterns.

### 3.2. Neurophysiological Properties of Uninjured (Left)-Injured (Right) Pairs of RS Neurons

At 2–3 wk recovery times following right spinal cord HTs at 10% BL, the passive properties, action potential features, and afterpotential characteristics of uninjured (left) and injured (right) pairs of the larger identified RS neurons (M2, M3, I1, B1, B3, B4; see Figure 1A) were compared (n = 38 left-right pairs of neurons, N = 27 animals; Table 3). This approach allowed comparisons for much of the data for uninjured-injured pairs of RS neurons in the same animal, tended to normalize for variations in properties of different neurons, and potentially reduced data variability (see Section 2.4 in Methods). The passive properties, AP features, afterpotential characteristics, and firing patterns (see below) of injured RS neurons will be referred to as the “injury phenotype”.

#### 3.2.1. Passive Electrical Properties

At 2–3 wk recovery times, some of the passive electrical properties for injured (right) RS neurons were significantly different compared to those for their paired uninjured (left) neurons (Table 3; paired *t*-tests, see Methods): (a) significantly more hyperpolarized resting membrane potential (V_REST_, *p* ≤ 0.05); (b) significantly longer membrane input time constant (τ_in_, *p* ≤ 0.001); and (c) significantly larger membrane input capacitance (C_in_, *p* ≤ 0.05). For membrane input resistance (R_in_), values for uninjured and injured pairs of neurons were not significantly different (*p* = 0.64).

#### 3.2.2. Action Potential Properties

At 2–3 weeks following right spinal cord HTs, several of the AP properties of injured (right) RS neurons were significantly different compared to those of their paired uninjured (left) neurons (Table 3, paired *t*-tests, see Methods): (a) significantly higher threshold voltage (ΔV_TH_, see Methods; *p* ≤ 0.001); (b) significantly higher threshold current (I_TH_; *p* ≤ 0.01; see Figure 3A,B); (c) significantly larger AP amplitude (V_AP_, *p* ≤ 0.01); (d) significantly longer AP duration (D_AP_, *p* ≤ 0.05); and (e) significantly larger slope for the repolarizing phase of APs (dV_m_/dt_fall_, *p* ≤ 0.05). Although V_REST_ was more hyperpolarized and V_AP_ was larger for injured RS neurons, the peaks of the APs were significantly more depolarization compared to those for uninjured neurons (*p* ≤ 0.05; paired *t*-test). For injured RS neurons, the significant increase in ΔV_TH_ was not due entirely to hyperpolarization of V_REST_ (Table 3) because V_TH_ was significantly more depolarized for injured neurons than for uninjured neurons (*p* ≤ 0.001, paired *t*-test; see Methods).

#### 3.2.3. Properties of Afterpotentials

In addition to changes in the main depolarization of APs, several changes occurred regarding the afterpotential components that followed APs (Table 3, Figure 3C). For example, for uninjured RS neurons, the repolarizing phase of APs usually was followed by three sequential afterpotentials (Figure 3(C1)): fAHP; ADP; and sAHP. Injured RS neurons displayed a significantly larger V_fAHP_ (*p* ≤ 0.001, unpaired *t*-test, see Methods) and significantly smaller V_ADP_ (*p* ≤ 0.001) and V_sAHP_ (*p* ≤ 0.001) compared to those for uninjured neurons (Figure 3(C2), Table 3), similar to that in previous reports [28,30]. For injured RS neurons, the increase in V_fAHP_ probably was due, in part, to an increase in the conductance of fast potassium channels (g_K_), since dV_m_/d_tfall_, which also depends on g_K_, increased significantly after injury (Table 3). Also, for injured RS neurons, the virtual absence of competing effects from the ADP and sAHP (Table 3) may have contributed to an apparent increase in V_fAHP_.

For injured RS neurons, the half-amplitude duration of the fAHP (D_fAHP_) was significantly longer (*p* ≤ 0.001, unpaired *t*-test, see Methods, Table 3; see Figure 3(C2)), and the delay of the fAHP (d_fAHP_) was significantly larger (*p* ≤ 0.001; see Figure 3(C2)) than those for uninjured neurons. Because the three afterpotential components probably overlap in time to some extent, the lengthening and increased delay of the fAHP for injured RS neurons probably resulted, in part, because of the virtual absence of competing effects from the ADP and sAHP (Table 3). In addition, the ~30% average increase in τ_in_ for injured RS neurons (Table 3 and Results above) probably contributed to the increase in D_fAHP_ and d_fAHP_. Finally, for injured RS neurons, durations (D) and delays (d) for the ADP and sAHP often could not be measured (n = 3, 4, or 6 in Table 3), and therefore, the statistical comparisons of these properties to those of uninjured neurons may not be meaningful.

#### 3.2.4. Repetitive Firing

For normal animals, 98% of RS neurons (n = 85/87 neurons; N = 28 animals) responded to above-threshold 2.0 s current pulses by firing a continuous train of APs, during which the spiking frequency usually smoothly decreased due to spike-frequency adaptation [32]. In addition, increasing the stimulus current from just above threshold to higher currents resulted in a progressive increase in firing rate, from relatively low spiking frequencies (1–5 Hz) to much higher frequencies (>50 Hz). In all cases, APs usually occurred during the entire depolarizing current pulse, except for certain RS neurons (e.g., M and I cells; see Figure 1A) that sometimes displayed delayed excitation (DE), which is a delay in firing at the onset of depolarization when preceded by a hyperpolarizing pre-pulse [32]. Delayed excitation is thought to be mediated by A-current [32].

At 2–3 wk recovery times following right spinal cord HTs, ~97% of uninjured (left) RS neurons (n = 35/37 neurons; N = 27 animals) displayed smooth firing during depolarizing current pulses (Figure 3(A1,B1)). Again, APs usually occurred continuously during the entire 2.0 s depolarizing current pulse (Figure 3(A1)), except for certain RS neurons that sometimes exhibited DE (arrowhead in Figure 3(B1)).

At 2–3 wk recovery times following right spinal cord HTs, ~92% of the injured (right) RS neurons (n = 33/36 neurons; N = 27 animals) displayed a single burst/single AP or short multiple bursts, characteristic of the “injury phenotype”, in response to depolarizing current pulses (Figure 3(A2,B2)), a slightly stronger response to SCI than described above (Table 1 and Table 2). Although SCI alters many of the biophysical properties of injured RS neurons (Table 3 and [28]), certain injured large RS neurons could still apparently express A-current and display DE (arrowhead in Figure 2(B2)). Interestingly, injured RS neurons usually exhibited membrane potential “resonance”, which was characterized as relatively high frequency (~15–30 Hz), low amplitude (~1–5 mV) oscillations of V_m_ during depolarization at or above threshold. Resonance was manifested as rapidly decaying V_m_ oscillations following a single burst of APs (inset in Figure 3(A2)) or growing V_m_ oscillations between multiple, short bursts of APs (inset in Figure 3(B2)) (Hough and McClellan, in preparation).

### 3.3. Dependency of Firing Patterns of Injured RS Neurons on Depolarizing Current Levels

**Figure 4:**
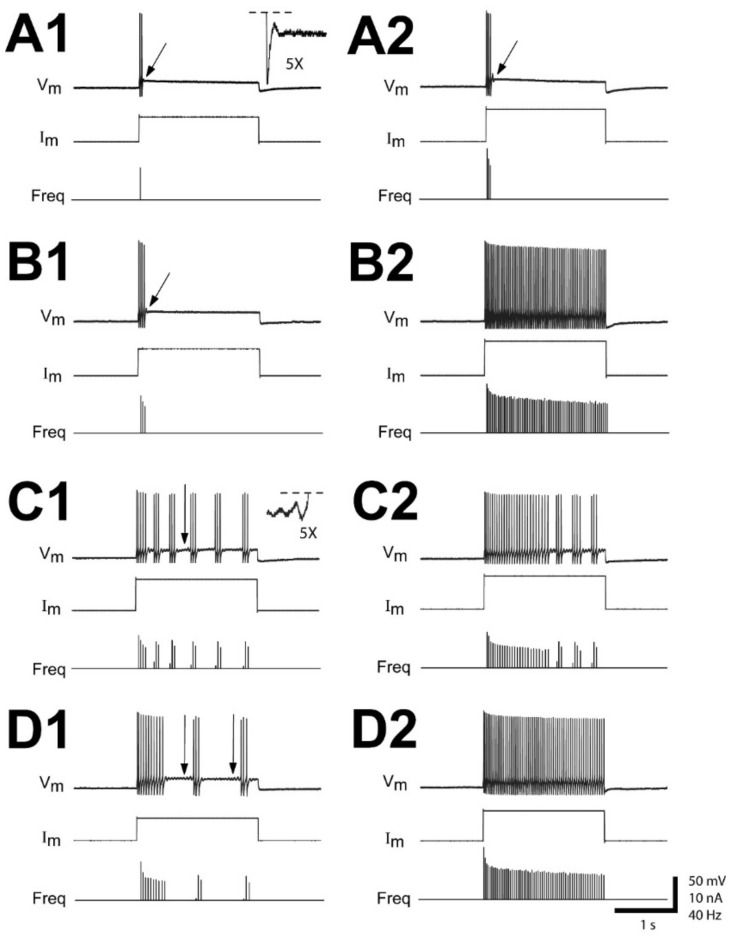
Variations in repetitive firing patterns with injected current levels. Recordings from injured (right) RS neurons in different animals at 2–3 wks following right spinal cord HTs at 10% BL (see Figure 1B). (**A**) Right “B3” neuron and (**B**) right “M3” neuron (see Figure 1A) that (**A1,B1**) initially fired a single burst in response to intermediate depolarizing current levels (see text), and at higher currents either (**A2**) continued to fire a single burst or (**B2**) fired continuously. Inset (5× enlargement) and arrows indicate “resonance”, which in this case was manifested by damped V_m_ oscillations following a single burst. (**C**) Right “I1” neuron and (**D**) right “B1” neuron that (**C1,D1**) initially fired multiple short bursts in response to intermediate depolarizing current levels (see text), and at higher currents either (**C2**) continued to fire multiple bursts or (**D2**) fired continuously. Inset (5× enlargement) and arrows indicate “resonance”, which in this case was manifested by progressively increasing V_m_ oscillations between multiple bursts. Horizontal dashed lines indicate truncated APs.

At 2–3 wk recovery times following right spinal cord HTs at 10% BL, the firing patterns of injured (right) RS neurons (n = 73 total neurons; N = 32 animals) sometimes depended on depolarizing current levels (Figure 4). At threshold current levels (4.8 ± 1.9 nA), injured RS neurons fired a single AP (82%), a single burst (15%), or multiple bursts (3%). For intermediate current levels, the firing patterns of injured RS neurons had a similar distribution as those shown in Table 1 and Table 2 (Figure 4(A1,B1,C1,D1)). At the highest current levels employed (~8–10 nA), 47% (n = 33/70) of injured RS neurons continued to display injury-type firing patterns (Figure 4(A2,C2)), while 53% (n = 37/70) could be made to fire continuously (Figure 4(B2,D2)) (note: 3 neurons did not fire even at the highest currents). Any of the largest injured RS neurons (M2, M3, I1, B1, B3, B4; see Figure 1A) could display injury-type firing patterns or fire continuously at the highest current levels. In addition, for those injured RS neurons that could be made to fire continuously at high current levels, at intermediate current levels, 53% (n = 20/37 neurons) fired a single burst and 47% (n = 17/37) fired multiple bursts. Accordingly, the firing patterns for injured RS neurons were categorized at intermediate current levels (Table 1 and Table 2). Nonetheless, the firing patterns for injured RS neurons were markedly different and distinct from those of uninjured neurons, which typically fired continuously for all depolarizing current levels at or above threshold (see above).

The membrane input resistances (R_in_) for injured RS neurons that displayed injury-type firing at the highest current levels were not significantly different from those that could be made to fire continuously (*p* = 0.42; unpaired *t*-test). In addition, injured neurons that exhibited injury-type firing patterns at the highest current levels and those that fired continuously both expressed resonance (arrows in Figure 4). However, the injured RS neurons that could be made to fire continuously had significantly lower current thresholds (I_TH_, 3.9 ± 1.3 nA; *p* ≤ 0.001) and voltage thresholds (ΔV_TH_, 15.3 ± 4.7 mV; *p* ≤ 0.05) than those for neurons that continued to display injury-type firing patterns at the highest depolarizing currents (6.4 ± 2.1 nA and 17.5 ± 3.5 mV, respectively) (unpaired *t*-tests). These differences may indicate some variation in the degree to which injured RS neurons respond to SCI (see Discussion).

### 3.4. Do Injured Ascending Pathways Contribute to the Injury Phenotype of RS Neurons?

**Figure 5:**
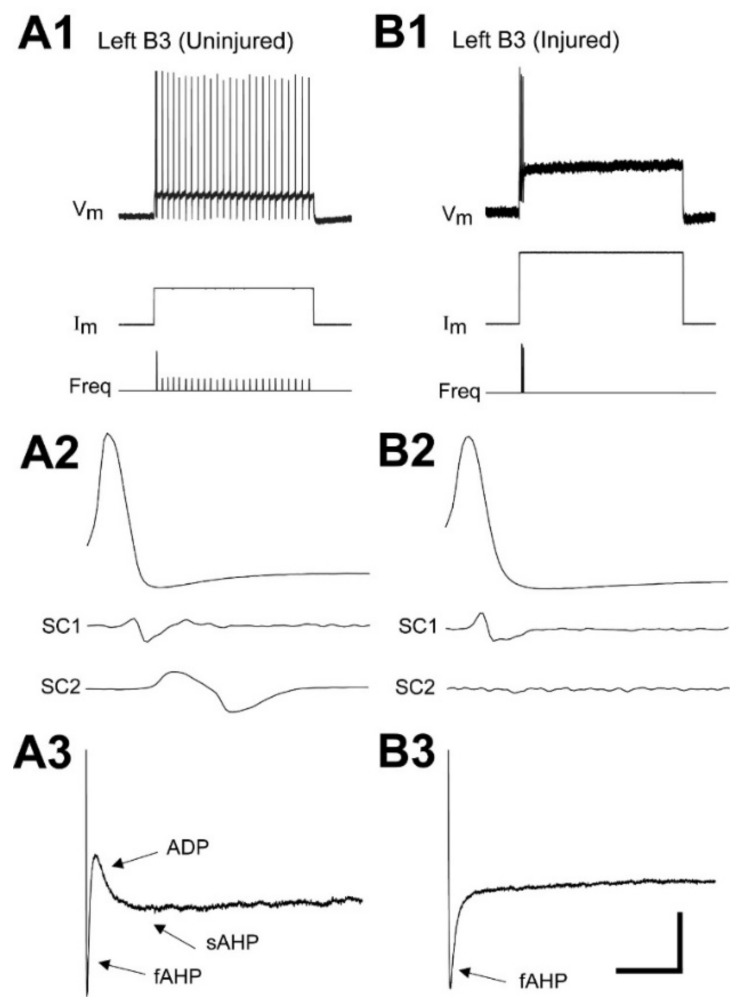
Interruption of ascending spinal-brain pathways does not contribute substantially to induction of injury-type firing patterns. Activity of (**A**) uninjured “B3” and (**B**) injured “B3” RS neurons (see Figure 1A) in different animals at 2 wks following lesions of the lateral spinal tracts and dorsal columns at 10% BL, which interrupt most ascending spinal-brain pathways (see Methods). (**A1,B1**) Membrane potential (V_m_), injected current (I_m_), and instantaneous firing (spiking) frequency (Freq) during applied 2.0 s depolarizing current pulses. (**A2,B2**) Evoked action potentials (top traces) and recordings from the spinal cord electrodes (SC1, SC2; see Methods) showing (**A2**) orthodromic responses rostral (SC1; see Figure 1B) and caudal (SC2) to the spinal lesion site for the uninjured neuron, and (**B2**) only a response rostral to the lesion site (SC1) for the injured neuron. (**A3,B3**) Afterpotentials following the repolarizing phase of action potentials: fAHP; ADP; and sAHP. Vertical/horizontal scale bar = (**A1,B1**) 50 mV, 7.5 nA, 67 Hz/875 ms; (**A2,B2**) 44.2 mV/2 ms; (**A3,B3**) 4.4 mV/47 ms.

At 2–3 wk recovery times following right spinal cord HTs at 10% BL (Figure 1B), the spinal lesion not only injured the descending axons of large, identified right RS neurons, which have *ipsilateral* descending axons [23,29], but also interrupted ascending spinal-brain pathways on the right side of the spinal cord. In contrast, complete spinal cord transections interrupt all descending and ascending pathways. Thus, following right spinal cord HTs, partial interruption of ascending spinal cord-brain pathways potentially might contribute to the “injury phenotype” of injured RS neurons. To test this possibility, animals received a lesion at 10% BL of the lateral spinal tracts (i.e., left and right lateral one-third of cord) as well as the dorsal columns (dorsomedial spinal cord) (n = 19 neurons, N = 4 animals), which together contain many of the ascending spinal pathways in the lamprey [39,40,41]. At 2–3 wk recovery times following these spinal lesions, all of the 13 uninjured RS neurons, which elicited orthodromic responses below the injury site (Figure 5(A2); see Figure 1B), displayed smooth, continuous repetitive firing (Figure 5(A1)) and all three afterpotential components following APs (fAHP, ADP, sAHP; Figure 5(A3)). In contrast, all of the 6 injured RS neurons, which did not elicit orthodromic responses below the injury site (Figure 5(B2)), exhibited injury-type repetitive firing (Figure 5(B1)) and only the fAHP afterpotential component following APs (Figure 5(B3)). The spinal lesions for these experiments were largely symmetrical and undoubtedly interrupted many of the ascending projecting axons, yet only RS neurons that were injured displayed the injury phenotype. Thus, the expression of the injury phenotype for right RS neurons following right spinal cord HTs appears to depend on neuronal injury of descending axons and not appreciably on the interruption of ascending spinal cord-brain pathways.

### 3.5. Contributions of the sAHP to Firing Patterns of Uninjured and Injured RS Neurons

**Figure 6:**
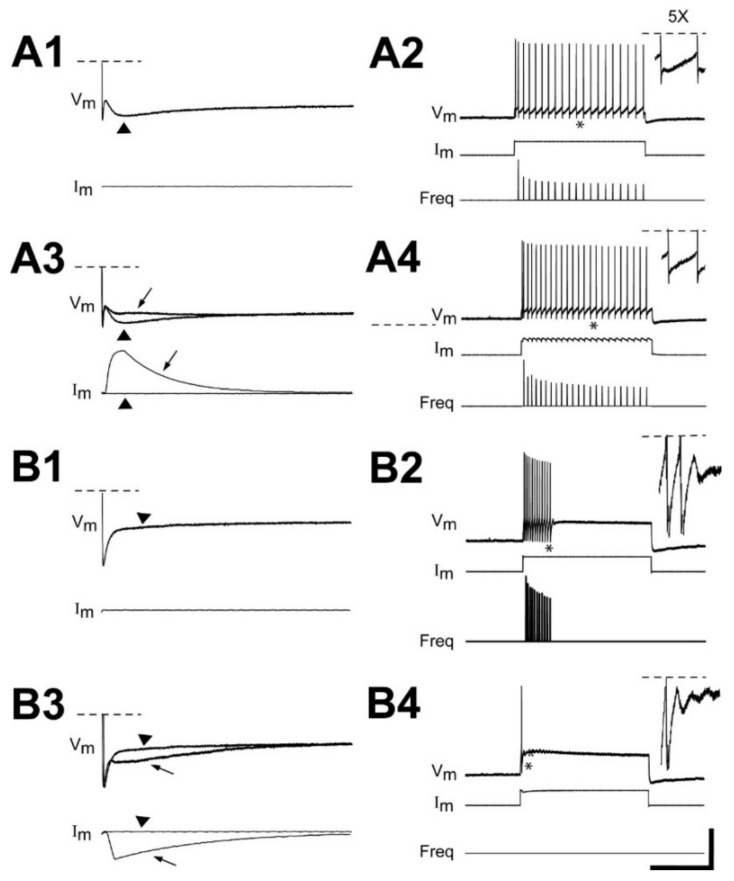
For injured RS neurons, the absence of the sAHP does not induce injury-type firing patterns. Recordings from (**A**) uninjured and (**B**) injured RS neurons in different animals that had recovered for 2 wks following a right spinal cord HT at 10% BL (see Figure 1B). (**A**) Uninjured left “B1” RS neuron (see Figure 1A). (**A1**) Recording of sAHP (arrowhead) following the repolarizing phase of a single action potential (AP), and (**A2**) continuous firing in the presence of the sAHP. Membrane potential (V_m_), injected current (I_m_), and instantaneous firing frequency (Freq). (**A3**) Afterpotentials before (arrowhead) and after (arrow) electronic removal of the sAHP waveform by current injection (I_m_, arrow; see Methods) following an AP, and (**A4**) higher repetitive frequency firing during 2.0 s depolarizing current pulses when the sAHP waveform was removed by current injection after each AP. (**B**) Injured right “M2” RS neuron. (**B1**) Lack of the sAHP (arrowhead) following a single AP and (**B2**) firing of a relatively short burst of APs in the absence of the sAHP. (**B3**) Afterpotentials before (arrowhead) and after (arrow) the addition of an sAHP waveform by current injection following a single AP, and (**B4**) reduced firing when the sAHP waveform was added after each AP. (**A2**,**A4**,**B2**,**B4**) Insets are expanded (5×) sections of recordings at * between or after APs. (**A**,**B**) Horizontal dashed lines indicate truncated APs (for **B2** and **B4**, fAHPs are truncated). Vertical/horizontal scale bars = (**A1,A3,B1,B3**) 10 mV, 0.5 nA/100 ms; (**A2**,**A4**,**B2**,**B4**) 50 mV, 8 nA, 20 Hz/1 s.

The sAHP contributes to the spiking frequencies of uninjured lamprey neurons [33,34]. For injured RS neurons, the absence of the sAHP compared to its presence for uninjured neurons (Figure 3C, Table 3) might contribute to the differences in repetitive firing patterns for these two types of neurons (Figure 3A,B). However, in animals that recovered for 2–3 wks following right spinal cord HTs, for uninjured (left) RS neurons (n = 6 neurons; N = 5 animals), injecting a current waveform to remove the sAHP following each AP (Figure 6(A1,A3); see Methods) simply increased the frequency of repetitive firing in response to 2.0 s depolarizing current pulses (Figure 6(A2,A4)), similar to the effects of blocking the sAHP with drugs [33,34]. For injured (right) RS neurons (n = 5), injecting a current waveform to introduce an sAHP following each AP (Figure 6(B1,B3)) reduced firing (Figure 6(B2,B4)). Thus, the virtual absence of the sAHP for injured RS neurons did not appear to be responsible for converting these neurons to injury-type firing patterns (i.e., single burst or multiple bursts) but probably contributed to overall spiking frequencies.

### 3.6. Excitability of Uninjured vs. Injured RS Neurons

**Figure 7:**
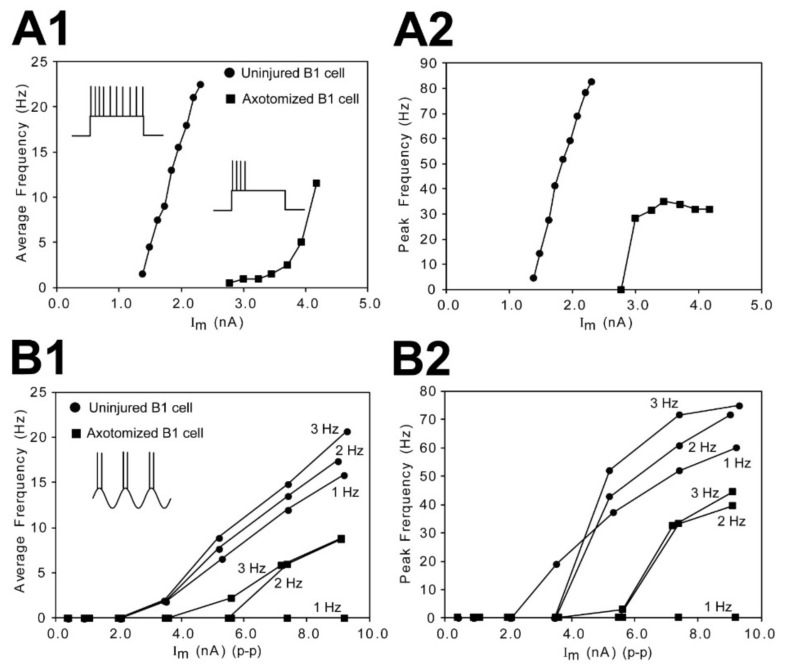
Injured RS neurons have lower excitability and lower spiking frequencies than uninjured neurons. Repetitive spiking frequencies for an uninjured (left) “B1” RS neuron (filled circles, ●) and injured (right) “B1” RS neuron (filled squares, ■) in the same brain from an animal that had recovered for 2 wks following a right spinal cord HT at 10% BL (see Figure 1B). (**A**) During applied 2.0 s depolarizing current pulses (I_m_), the injured RS neuron fired at (**A1**) lower average and (**A2**) lower peak spiking frequencies (■) than those for the uninjured neuron (●). This particular injured neuron fired a single burst at the onset of a depolarizing current pulse. (**B**) During applied sinusoidal current injection (I_m_; p-p = peak-to-peak) at 1, 2 or 3 Hz, the (**B1**) average and (**B2**) peak spiking frequencies for the injured RS neuron were lower (■) than those for the uninjured neuron (●).

At 2–3 wk recovery times following right spinal cord HTs at 10% BL, injured RS neurons had significantly higher current thresholds (I_TH_) and voltage thresholds (ΔV_TH_) for eliciting action potentials (Table 3). Accordingly, at these recovery times, the data suggest that injured (right) RS neurons should have lower excitability than uninjured (left) neurons. Based on recordings of uninjured (left)-injured (right) pairs of RS neurons, this appears to be the case. First, in response to 2.0 s depolarizing current pulses (Figure 7A; n = 43 left-right pairs of neurons, N = 33 animals), injured RS neurons had significantly lower average spiking frequencies for 35/43 pairs of neurons (81% of pairs; *p* < 0.001, one-tail Sign test; see Methods) and significantly lower peak spiking frequencies for 32/43 pairs of neurons (74%; *p* = 0.001) for the same corresponding depolarizing current values. Second, for injection of sinusoidal current waveforms (Figure 7B; n = 14 left-right pairs of neurons, N = 12 animals), average spiking frequencies for 10/14 pairs of RS neurons (71% of pairs; *p* = 0.09) and peak spiking frequencies for 10/14 pairs of neurons (*p* = 0.09) were lower for injured compared to those for uninjured RS neurons, although the *p* values were not quite significant, possibly because of comparatively lower n values. Interestingly, some injured RS neurons fired in response to 2 or 3 Hz sinusoidal current waveforms but not 1 Hz waveforms (Figure 7B) (Hough and McClellan, in preparation). Thus, for a given applied above-threshold depolarizing current, injured RS neurons had substantially lower excitability and lower spiking frequencies compared to uninjured neurons.

### 3.7. Sensory-Evoked Synaptic Responses for Uninjured and Injured RS Neurons

For the lamprey, contralateral or ipsilateral stimulation of the lateral oral hood elicits synaptic responses in RS neurons [37,38] that are thought to be mediated via trigeminal sensory neurons [42,43]. These responses involve a disynaptic pathway through second-order sensory neurons in the nucleus of the descending tract of the trigeminal nerve [43].

#### 3.7.1. Synaptic Potential Types

For RS neurons in normal animals and animals with right spinal cord HTs, contralateral or ipsilateral stimulation of the lateral oral hood elicited “monophasic EPSPs” in ~82% of the neurons (n = 31/38 neurons, N = 18 animals). For 16% of the neurons (n = 6/38), the synaptic responses were mixed PSP-EPSPs for which the major component was the EPSP. The amplitudes of EPSPs and the EPSP component of PSP-EPSPs were measured, and these two types of synaptic responses will be referred to as “EPSPs”. An IPSP response was elicited for only one uninjured RS neuron (n = 1/38 neurons ~2%), which was not included in the analysis below.

#### 3.7.2. Contralaterally-Evoked vs. Ipsilaterally-Evoked EPSPs for RS Neurons

**Figure 8:**
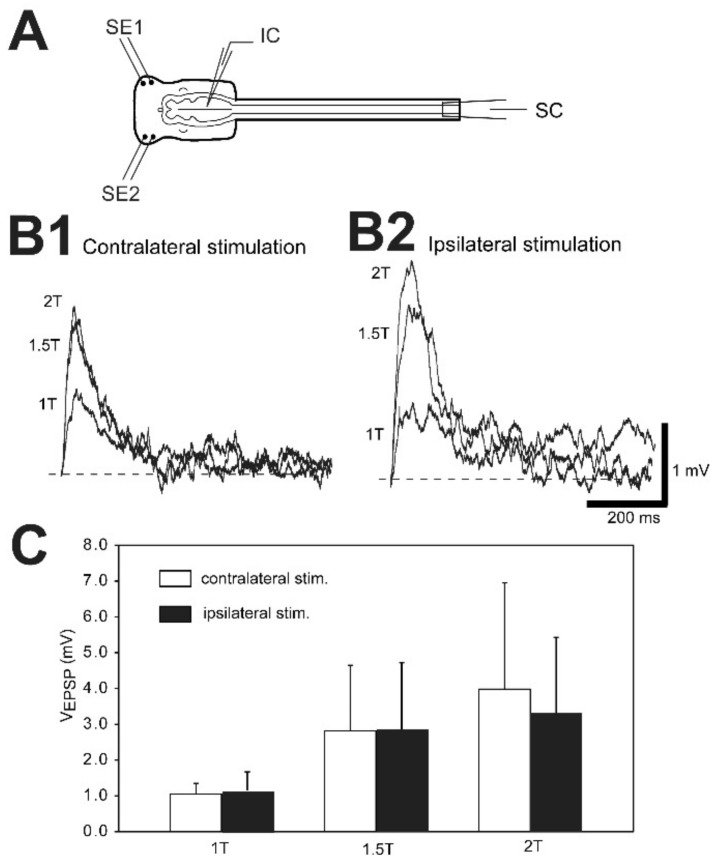
Comparison of contralateral vs. ipsilateral sensory-evoked EPSPs for RS neurons in normal animals. (**A**) Diagram of in vitro brain-spinal cord preparation (not to scale) from a normal animal showing exposed brain (right) and spinal cord (left), intracellular micropipette (IC) for recording from RS neurons, suction electrode (SC) for recording RS neuron-evoked orthodromic action potentials in the spinal cord, and right and left oral hood stimulating electrodes (SE1 and SE2). (**B1**) Traces of averaged EPSPs evoked in a left “B3” RS neuron (see Figure 1A) by contralateral stimulation of the lateral oral hood (SE1) at various intensities (1 T, 1.5 T and 2 T; see Methods). Horizontal dashed lines indicate V_REST_. (**B2**) Traces of EPSPs evoked in the same left “B3” neuron by ipsilateral stimulation of the lateral oral hood (SE2) at various intensities. (**C**) Average amplitudes (bars = means; vertical lines = SDs) of EPSPs recorded from RS neurons evoked by stimulation of the contralateral (open bars) or ipsilateral (filled bars) oral hood at different stimulus intensities (n = 20 RS neurons, N = 9 animals). For each stimulus intensity (1 T, 1.5 T, and 2 T), the contralaterally-evoked and ipsilaterally-evoked EPSP averages were not significantly different (*p* = 0.45, 0.95, and 0.47, respectively; unpaired *t*-test, with Welch correction when appropriate; see Methods).

For normal animals, sensory-evoked EPSPs recorded from RS neurons were compared for contralateral and ipsilateral stimulation at different oral hood stimulation intensities (1 T, 1.5 T and 2 T; see Methods; Figure 8A; n = 20 neurons, N = 9 animals). First, increasing the oral hood stimulus intensity resulted in an increase in evoked EPSP amplitudes by an average factor of ~2.5–3.5 (1 T to 1.5 T) and ~1.4 (1.5 T to 2 T) for either contralateral or ipsilateral stimulation (Figure 8B). Increases in stimulation intensity from 1 T to 1.5 T resulted in a substantial increase in EPSP amplitudes (>20% increase) for ~84% (31/37) of the contralateral and ipsilateral stimulation tests. In contrast, for increases from 1.5 T to 2 T, ~58% (21/36) of the contralateral and ipsilateral stimulation tests resulted in only a modest increase in EPSP amplitudes (<20% increase). Second, at oral hood stimulus intensities of 1.5 T or 2 T, ~8–10% of neurons produced APs, making paired statistical comparisons of EPSP amplitudes sometimes problematic. Third, at each stimulus intensity (1 T, 1.5 T, and 2 T), the EPSP amplitudes evoked by contralateral oral hood stimulation were not significantly different compared to those for ipsilateral stimulation (Figure 8C; *p* = 0.45, 0.95, and 0.47, respectively; unpaired *t*-tests, with Welch correction when appropriate; see Section 2.5 in Methods).

#### 3.7.3. Sensory-Evoked EPSPs for Injured and Uninjured Neurons

**Figure 9:**
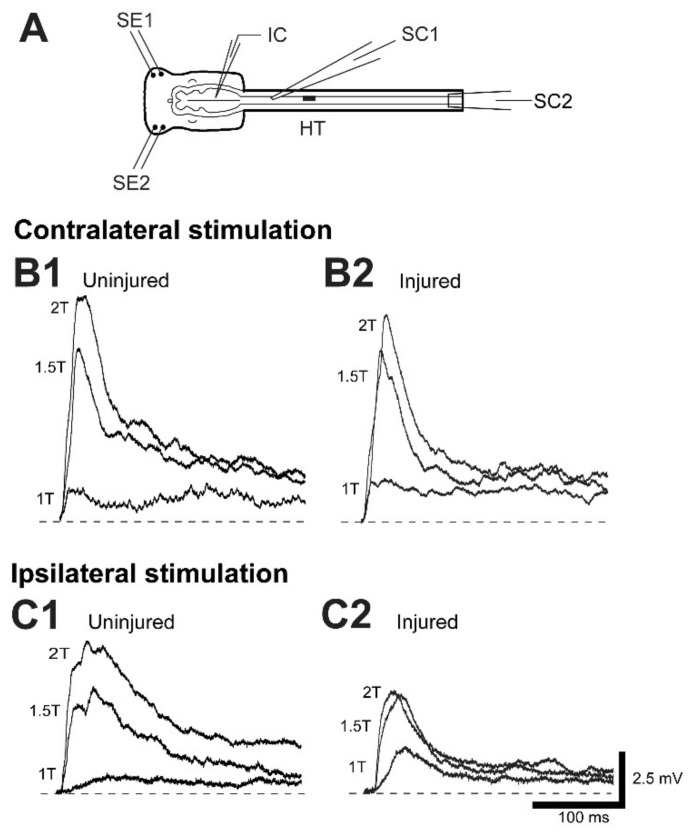
Comparison of sensory-evoked EPSPs recorded from uninjured and injured pairs of RS neurons. (**A**) Diagram of in vitro brain-spinal cord preparation (not to scale) showing the exposed brain (right) and spinal cord (left), right spinal cord HT at 10% BL (2 wk recovery time), intracellular micropipette (IC) for recording from RS neurons, suction electrodes for recording RS neuron-evoked orthodromic action potentials in the spinal cord (SC1 and SC2; see Figure 1B), and right and left oral hood stimulating electrodes (SE1 and SE2). (**B**) Traces of averaged EPSPs for (**B1**) an uninjured left “B1” RS neuron and (**B2**) injured right “B1” RS neuron (see Figure 1A) in the same animal evoked by contralateral oral hood stimulation of varying stimulus intensities (1 T, 1.5 T, 2 T; see Methods). (**C**) Traces of averaged EPSPs for (**C1**) an uninjured left “B3” RS neuron and (**C2**) injured right “B3” RS neuron in the same animal evoked by varying stimulus intensities applied to the ipsilateral oral hood. Horizontal dashed lines indicate V_REST_.

First, for animals that had recovered for 2–3 wks following right spinal cord HTs at 10% BL (Figure 9A), R_in_ values for uninjured and injured pairs of RS neurons (n = 11 left-right pairs; N = 10 animals) were not significantly different (*p* = 0.94, unpaired *t*-test), verifying the earlier results in Table 3. Second, oral hood stimulation at 1.5 T or 2 T sometimes elicited action potentials in uninjured or injured RS neurons, making paired statistical comparisons sometimes problematic. Third, increasing oral hood stimulation intensity from 1 T to 1.5 T usually substantially increased (>20% increase) the evoked EPSP amplitudes (Figure 9B,C), while increasing stimulus intensity from 1.5 T to 2 T often resulted in only modest increases in EPSP amplitudes (<20% increase), similar to the results above for RS neurons in normal animals.

**Figure 10:**
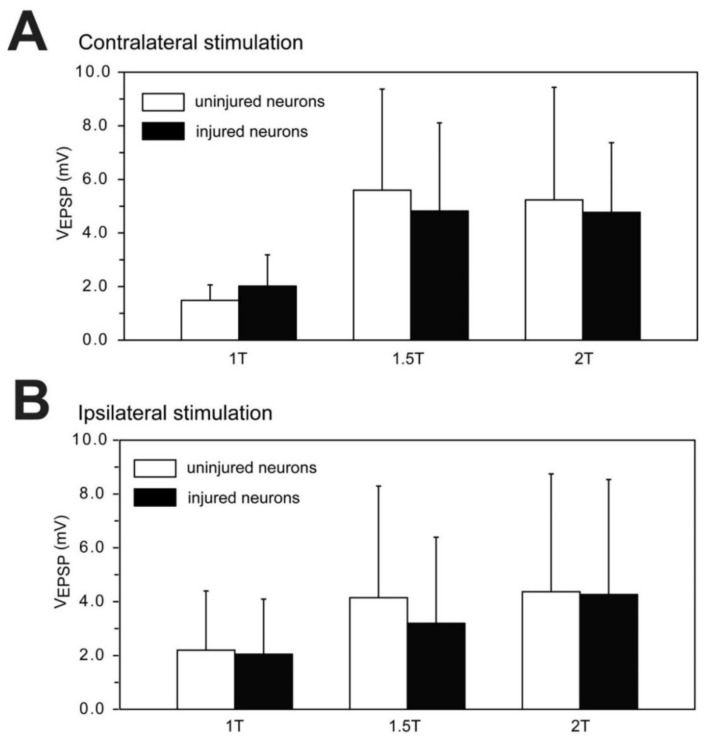
Amplitudes of sensory-evoked EPSPs are similar for uninjured and injured RS neurons. Average amplitudes (bars = means; vertical lines = SDs) of oral hood-evoked EPSPs for uninjured and injured pairs of RS neurons (n = 11 left-right pairs of RS neurons, N = 10 animals). (**A**) Average EPSP amplitudes evoked in uninjured (open bars) and injured (filled bars) RS neurons by contralateral stimulation of the oral hood at different stimulus intensities (1 T, 1.5 T, 2 T; see Methods). (**B**) Average EPSP amplitudes evoked by ipsilateral stimulation of the oral hood at different stimulus intensities. At each stimulus intensity for either contralateral and ipsilateral stimulation, the EPSP amplitudes were not significantly different for uninjured and injured RS neurons (*p* = 0.20–0.94, see text; unpaired *t*-test, with Welch correction when appropriate; see Methods).

At each oral hood stimulus intensity (1 T, 1.5 T, and 2 T) for contralateral stimulation, the evoked EPSP amplitudes were not significantly different between uninjured and injured RS neurons (Figure 10A; *p* = 0.20, 0.63, and 0.94, respectively; unpaired *t*-tests, with Welch correction when appropriate; see Section 2.5 in Methods). Likewise, for ipsilateral stimulation, at each of the stimulus intensities (1 T, 1.5 T, and 2 T), the evoked EPSP amplitudes were not significantly different between uninjured and injured RS neurons (Figure 10B; *p* = 0.84, 0.43, and 0.94, respectively). Thus, at 2–3 wk recovery times, SCI did not appear to substantially alter trigeminal sensory-evoked synaptic responses recorded from injured RS neurons. In addition, the decrease in excitability displayed by injured RS neurons did not appear to be compensated for by an increase in sensory-evoked synaptic response for these neurons, at least for trigeminal sensory inputs.

## 4. Discussion

### 4.1. Injury Phenotype for Lamprey RS Neurons Following SCI

Previous studies had demonstrated that following SCI for the lamprey, injured B cells (see Figure 1A) displayed several dramatic changes in their properties, described as the “injury phenotype”, compared to uninjured neurons [28]: (a) altered firing patterns; (b) changes in afterpotential components; and (c) down-regulation of SK channels and HVA calcium channels. The data from the present study greatly extended the above findings regarding the underlying neuronal mechanisms for the “injury phenotype”, as described below.

First, it was established that following rostral spinal cord HTs, injured RS neurons began to display changes in firing patterns in as little as ~2–3 days, the altered firing patterns were maximally displayed at 2–3 wks, firing patterns then gradually recovered, and by 12–16 wks firing patterns were back to normal for the majority of neurons (~70%). Except for M3, which is the poorest axonal regenerator among Müller cells [18] and accounted for only ~5% of neurons sampled at 12–16 wk recovery times, the percentages of the other different RS neurons that were recorded from at different recovery times were roughly comparable (see Table A1 in Appendix A). Müller cells, particularly those with poorer regenerative capacities [18], can sometimes undergo apoptosis at longer recovery times [44], and of course these cells were not included in our analysis. However, except for M3, this phenomenon did not appear to cause an obvious sampling bias in our results (see Table A1 in Appendix A) because relatively few injured RS neurons were recorded from per brain (Table 1), and there always were several healthy Müller cells available to record from in each brain even though a few of these cells may have died. Finally, at longer recovery times, it is likely that the restoration of normal firing patterns for injured RS neurons was due, in part, to these neurons forming synapses below the injury site to restore target-derived neurotrophic support [30].

Second, neurophysiological recordings from uninjured-injured pairs of RS neurons indicated that compared to uninjured neurons, injured neurons displayed several significant changes in their biophysical properties at 2–3 wk recovery times following spinal cord HTs (Table 3): (a) more hyperpolarized V_REST_; (b) longer membrane input time constant (τ_in_) and larger membrane input capacitance (C_in_); (c) increase in voltage thresholds (ΔV_TH_) and current thresholds (I_TH_); (d) larger amplitude (V_AP_) and duration (D_AP_) of action potentials (APs); (e) higher slope for the repolarizing phase of APs (dV_m_/dt_fall_); (f) larger V_fAHP_, D_fAHP_, and d_fAHP_; (g) near abolishment of the ADP and sAHP; and (h) lower average and peak spiking frequencies during applied depolarization. Following SCI, the increase in thresholds, which will make injured RS neurons less likely to fire, might be a partial compensatory response to the near abolishment of the sAHP, which will increase spiking frequencies.

Third, at 2–3 wk recovery times, the majority of injured RS neurons displayed injury-type firing patterns at intermediate depolarizing current levels: single, short burst/single AP; or multiple short bursts. However, some injured RS neurons could fire continuously at relatively high currents. These differences as well as the general variability in the injury-type firing patterns of injured RS neurons (Table 2) may indicate some variation in the degree to which these neurons respond to SCI. The induction of injury-type firing patterns for injured RS neurons did not appear to be dependent on disruption of ascending spinal-brain pathways or the virtual absence of the sAHP.

Fourth, at 2–3 wk recovery times, trigeminal sensory-evoked synaptic responses for injured RS neurons were not substantially altered compared to those for uninjured neurons. Thus, the decrease in excitability of injured RS neurons does not appear to be compensated for by an increase in sensory-evoked synaptic response for these neurons, at least for trigeminal sensory inputs. However, synaptic input differences, if present, might occur at longer recovery times or in response to non-trigeminal sensory inputs [42]. Also, at 2–3 wk recovery times, the ~30% significant increase in C_in_ (see Table 3) suggests that the surface area of injured RS neurons may have increased, although this could have been due to membrane enfolding and not enlargement of soma diameters. In the future, it will be important to inject uninjured and injured pairs of RS neurons with fluorescent dyes and compare their morphologies in detail at multiple recovery times using confocal microscopy.

### 4.2. Contribution of Injury Phenotype to Axonal Regeneration of Lamprey RS Neurons

For injured RS neurons, some of the above changes in biophysical properties and firing patterns that are part of the injury phenotype are expected to provide supportive conditions for axonal regeneration. First, for lamprey RS neurons in culture, experimentally evoked increases in calcium influx (via voltage-gated and/or ligand-gated calcium channels) resulted in inhibition of neurite outgrowth or neurite retraction, and blocking calcium influx negates these effects [45], similar to the results from studies of other neurons (reviewed in [46,47,48]). Thus, increases in intracellular calcium might be inhibitory for axonal regeneration of lamprey RS neurons following SCI. Second, at 2–3 wk recovery times following right spinal cord HTs at 10% BL, right reticular nuclei, which contained mostly injured RS neurons, exhibited a reduction in expression of mRNA for HVA calcium and SK channels compared to left nuclei, which contained mostly uninjured RS neurons (also see [23,28]). Lastly, the present study demonstrated a significant hyperpolarization of V_REST_ and a reduction in voltage and current thresholds (Table 3) as well as a decrease in excitability and spiking frequencies of injured RS neurons compared to uninjured neurons (Figure 7). Together, these results suggest that following SCI, the down regulation of Ca^++^ channels and reduction of excitability for injured RS neurons would be expected to reduce calcium influx, particularly during electrical activity, and maintain intracellular calcium levels in a range that is supportive for axonal regeneration ([28,45]; reviewed in [15]). This hypothesis that reduced calcium influx is important for axonal regeneration of injured lamprey RS neurons following SCI is supported by data from mammalian neurons [49,50,51]. Interestingly, blocking calcium channels for *uninjured* lamprey RS neurons causes these neurons to display injury-type firing patterns [28]. Finally, many axotomized peripherally-projecting neurons, whose peripheral axons generally are capable of axonal regeneration, exhibit a reduction in calcium currents, conductances, and/or channel expression levels (e.g., [52,53]; reviewed in [28]).

### 4.3. Comparisons to Other Studies of Lamprey Neurons Following SCI

At ~8-12 wks after SCI at ≥10 mm caudal to the cell bodies of large, identified lamprey RS neurons (Müller cells), it was stated that the somata were swollen, and V_REST_ often was reduced by 30–40 mV (summarized in [54]). For 8–16 wk recovery times, the majority of injured RS neurons that were visible and could be recorded from were healthy (i.e., good V_REST_ and AP properties), provided they were allowed to regenerate their axons ([28]; Figure 2(A6) in present study). However, when axonal regeneration of these neurons was physically blocked for 8 wks following SCI, large RS neurons often appeared swollen and had poor electrical properties [30,55], presumably because they could not make synapses below the injury site to restore target-derived neurotrophic support [30]. At ~8–12 wks after SCI, it was stated that the dendritic trees of large lamprey RS neurons were reduced, and many of the synapses on the dendrites of these neurons were lost (summarized in [54]). We did not observe neurophysiological correlates of these suggested synaptic changes at 2–3 wk recovery times (Figure 9 and Figure 10). In addition, at 8–12 wk recovery times following SCI, recovered lampreys did not display apparent changes in threshold or display deficits for trigeminal sensory-evoked flexion and swimming responses [16], during which large, identified RS neurons are activated in normal animals [25,26,43].

At ~7 wk recovery times following axotomy that was <500 μm from the cell bodies of large lamprey RS neurons (i.e., lesion within the brainstem), these neurons mainly exhibited dendritic sprouting and an increase in soma diameter, while axotomy in the spinal cord ~10–20 mm caudal to their cell bodies mainly elicited axonal sprouting [56,57]. Axotomy at intermediate distances (~1000–1400 μm) from the cell bodies evoked both dendritic and axonal sprouting [57].

At 4–5 wks following SCI in the lamprey, injured spinal dorsal cells (DCs; centrally located primary sensory neurons) displayed a significant increase in R_in_, ΔV_TH_, I_TH_, and D_AP_, and a significant decrease in dV_m_/dt_rise_ and V_REST_ [58]. Also, at ~3 wk recovery times, DCs exhibited a significant decrease in cell diameter [58]. However, injured lamprey DCs usually exhibit limited regeneration of their ascending axons [59], and thus, some of the changes mentioned above may reflect this aspect of their response to injury. Also, the average recorded V_REST_ values for *uninjured* DCs were more positive than −60 mV and for *injured* DCs were more positive than −50 mV [58], while other uninjured lamprey spinal neurons have average V_REST_ values more negative than −75 mV [60]. In contrast to lamprey DCs, giant interneurons (GIs) and lateral cells (LCs), both of which are spinal interneurons, exhibited an increase in cell diameter following SCI, sometimes for up to 20 wks post-transection, and a simplification in their dendritic trees [61]. In addition, there were changes in the relative occurrence of spontaneous EPSPs and IPSPs for these neurons, and a decrease in V_REST_.

### 4.4. Biophysical and Morphological Properties of Injured Neurons in Other Animals

For vertebrates, changes in biophysical properties of injured (axotomized) peripherally-projecting neurons are quite variable and sometimes difficult to generalize. For example, axotomized cat motoneurons (MNs) displayed an increase in R_in_, D_AHP_, and τ_in_, and a decrease in conduction velocity [62]. Axotomy of sympathetic neurons (SNs) in the bullfrog resulted in an increase in D_AP_ and dV_m_/dt_fall_ and a decrease in V_AHP_ and D_AHP_, and there was a partial restoration of normal properties upon reinnervation with peripheral targets [63,64]. Injured rat SNs exhibited a decrease in V_REST_, V_AP_, D_fAHP_, τ_in_ and R_in_ [65].

In some cases, experimental results were variable between studies of the *same* type of neuron but in *different* types of animals. For example, in cat and rat, injured dorsal root ganglia (DRG) neurons displayed a decrease in the amplitude and duration of the AHP [66,67], while the duration of AHPs for injured DRG neurons in hamster [68] or mouse [69] did not change. In other cases, results were variable between studies of the *same* type of neuron in the *same* type of animal. For example, following peripheral axonal injury of rat DRG neurons, V_REST_ increased [70], decreased [71], or did not change [52], and V_AP_ increased [52], decreased [70], or did not change [72]. Also, for injured rat DRG neurons, R_in_ decreased slightly [70] or increased [73]. Following axotomy of cat spinal MNs, the results also are somewhat variable for different studies. Some of the variability of the above results between the different studies may be due to differences in techniques, particularly the method for performing lesions.

Following injury, vertebrate interneurons appear to display less variable changes in biophysical properties than peripherally projecting neurons (MNs, SNs, DRG neurons). For example, injured descending brain neurons (i.e., corticospinal or rubrospinal) exhibited no change in V_REST_, V_AP_, or D_AP_, and an increase in R_in_ [74,75].

The most comparable study to the present one involves the response of the Mauthner cell in goldfish to SCI at ~7–10 mm caudal to the cell bodies of these neurons. At ~3–5 wk recovery times, this descending brain neuron exhibited no change in V_REST_ or R_in_, but an increase in V_AP_ [76] and an increase in cell diameter [77].

In contrast to the *decrease* in excitability of injured RS neurons demonstrated in the present study, axotomy usually resulted in an *increase* in excitability for injured spinal MNs [62], DRG neurons [69], and goldfish Mauthner cells [76]. For DRG neurons, this increase in excitability can result in spontaneous V_m_ oscillations at V_REST_ that appear to contribute to neuropathic pain [69]. In addition, axotomy of MNs often resulted in a reduction of synaptic inputs from Ia afferents [78,79] that appears to be due, in part, to retraction of the MN dendritic tree (i.e., “dendritic stripping”; reviewed in [80]). Dendritic stripping also occurs for axotomized rubrospinal neurons [81] and superior cervical ganglion neurons [82], but in the latter case, reinnervation with peripheral targets restores dendritic morphology.

## 5. Conclusions

In the present study, the time course for altered repetitive firing patterns for injured lamprey RS neurons following SCI was determined, and the passive properties, action potential features, afterpotential characteristics, repetitive firing properties, and trigeminal sensory-evoked synaptic responses were compared for uninjured-injured pairs of RS neurons. In particular, following SCI, injured RS neurons displayed significantly higher voltage thresholds (ΔV_TH_) and current thresholds (I_TH_) than uninjured neurons and had lower excitability, as indicated by a decrease in average and peak spiking frequencies during applied depolarization. Because HVA calcium channels appear to be down regulated in RS neurons following SCI (McClellan et al., 2008), a concurrent reduction in excitability of these neurons would be expected to reduce calcium influx and provide a supportive intracellular environment for axonal regeneration.

## Figures and Tables

**Table 1:** Firing Patterns of Injured Reticulospinal (RS) Neurons ^a^ at Different Recovery Times following Right Spinal Cord Hemi-Transections (HTs) at 10% Body Length (BL, normalized distance from anterior tip of head).

**Recovery Time**	**Single Short Burst** **or Single AP**	**Multiple** **Short Bursts**	**Irregular**	**Smooth**
**2–3 days (n = 13 neurons)** **(N = 4 animals)**	8% ^b^ (0/1) ^c^	0%	69% (0/9)	23% (0/3)
**1 wk (n = 15)** **(N = 7)**	27% (0/4)	46% (0/7)	27% (1/4)	0%
**2–3 wks (n = 145)** **(N = 53)**	46% (0/67)	30% (2/43)	13% (0/19)	11% (3/16)
**4 wks (n = 25)** **(N = 9)**	24% (0/6)	44% (0/11)	16% (1/4)	16% (0/4)
**6 wks (n = 33)** **(N = 16)**	49% (1/16)	15% (0/5)	9% (0/3)	27% (4/9)
**8 wks (n = 9)** **(N = 5)**	33% (0/3)	11% (0/1)	0%	56% (4/5)
**12–16 wks (n = 19)** **(N = 8)**	26% (3/5)	0%	5% (1/1)	69% (12/13)

a—large, identified injured (right) RS neurons (see text and Figure 1B): M cells; I cells; B cells (Müller cells, see Figure 1A); some of the B cell from [28] and [30] were used for the present analysis. b—percentage of RS neurons with a particular firing pattern at intermediate current levels (see rationale in Section 3.3 of Results). c—fraction of RS neurons with a particular firing pattern that elicited orthodromic responses caudal to right spinal cord HTs at 10% BL (see SC2 in Figure 1B).

**Table 2:** Firing Patterns of Different Large, Identified Injured RS Neurons ^a^ at 2–3 Week Recovery Times Following Right Spinal Cord HTs at 10% BL.

**RS Neuron**	**Single Short Burst** **or Single AP**	**Multiple** **Short Bursts**	**Irregular**	**Smooth**
**M2 (n = 24 neurons)**	42% ^b^	25%	17%	16%
**M3 (n = 19)**	58%	26%	0%	16%
**I1 (n = 17)**	47%	29%	12%	12%
**B1 (n = 32)**	47%	34%	13%	6%
**B3 (n = 25)**	52%	28%	16%	4%
**B4 (n = 25)**	36%	32%	20%	12%

a—total of n = 142 injured (right) large, identified RS neurons (Müller cells; see text and Figure 1); N = 53 animals (note: insufficient n values for M1, I2-I4, B2, B5); some of the B cell from [28] were further analyzed and used for the present study. b—percentage of RS neurons with a particular firing pattern at intermediate current levels (see rationale in Section 3.3 of Results).

**Table 3:** Biophysical Properties of Uninjured and Injured Pairs of RS Neurons ^a^ at 2–3 Week Recovery Times following Right Spinal Cord HTs at 10% BL.

**Passive Properties**						
	**V_rest_ (mV)**		**R_in_ (MΩ)**	**τ_in_ (ms)**		**C_in_ (nF)**
**Uninjured**	−72.20 ± 5.24 ^b^ (n = 38) ^c^		5.21 ± 2.82 (n = 36)	7.24 ± 3.35 (n = 35)		1.71 ± 1.00 (n = 35)
**Injured**	−74.72 ± 4.32 * (n = 38)		5.38 ± 2.84 (n = 36)	9.50 ± 4.49 *** (n = 35)		2.02 ± 1.08 * (n = 35)
**Action Potential Features**						
	**Δ** **V_TH_ (mV)**	**I_TH_ (nA)**	**V_AP_ (mV)**	**D_AP_ (ms)**	**dV_m_/dt_rise_ (mV/ms)**	**dV_m_/dt_fall_ (mV/ms)**
**Uninjured**	11.48 ± 4.89 (n = 36)	3.28 ± 1.62 (n = 37)	103.21 ± 10.18 (n = 37)	0.98 ± 0.12 (n = 36)	249.67 ± 54.47 (n = 36)	−135.08 ± 24.33 (n = 36)
**Injured**	18.07 + 5.73 *** (n = 36)	4.68 ± 2.46 ** (n = 37)	109.31 ± 8.45 ** (n = 37)	1.04 ± 0.17 * (n = 36)	254.48 ± 55.80 (n = 36)	−147.10 ± 28.33 * (n = 36)
**Afterpotential Properties**						
	**V_fAHP_ (mV)**	**D_fAHP_ (ms)**	**d_fAHP_ (ms)**	**V_ADP_ (mV)**	**D_ADP_ (ms)**	**d_ADP_ (ms)**
**Uninjured**	−5.50 ± 3.37 (n = 28)	3.67 ± 4.68 (n = 27)	2.14 ± 0.51 (n = 28)	2.41 ± 1.81 (n = 26)	9.94 ± 3.69 (n = 19)	6.75 ± 2.20 (n = 24)
**Injured**	−11.36 ± 3.04 *** (n = 37)	8.21 ± 4.15 *** (n = 37)	2.65 ± 0.43 *** (n = 37)	0.06 ± 0.21 *** (n = 33)	18.43 ± 10.03 (n = 4)	22.58 ± 2.98 *** (n = 4)
	**V_sAHP_ (mV)**		**D_sAHP_ (ms)**		**d_sAHP_ (ms)**	
**Uninjured**	−1.19 ± 0.98 (n = 35)		96.62 ± 20.37 (n = 24)		49.91 ± 28.68 (n = 32)	
**Injured**	−0.10 ± 0.28 *** (n = 34)		77.29 ± 55.24 (n = 3)		60.61 ± 14.32 (n = 6)	

a—38 uninjured (left)-injured (right) pairs of large, identified RS neurons (see text and Figure 1); N = 27 animals. b—mean ± SD. c—number of neurons for which a given parameter was measured. Note: (i) afterpotential amplitudes were sometimes not measurable (see Methods); (ii) certain afterpotential durations (D) or delays (d) could not be measured when a particular component was not measurable, or when the component amplitude was 0.0 mV or very small. Statistics: Passive properties and action potential properties for uninjured-injured pairs of RS neurons were compared with a paired *t*-test (see Methods); afterpotential properties for uninjured and injured RS neurons generally had very different n values and were compared with an unpaired *t*-test, with Welch correction when appropriate (see Methods): *—*p* ≤ 0.05, **—*p* ≤ 0.01, ***—*p* ≤ 0.001.

## Data Availability

Not applicable.

## Appendix A

**Table A1:** Percentages of Different Injured RS Neurons ^a^ in Table 1 that were Recorded from at Different Recovery Times following Right Spinal Cord HTs at 10% BL.

**Recovery Time**	**M2**	**M3**	**I1**	**B1**	**B3**	**B4**
**2–3 days** **(n = 13 neurons)**	0% ^b^	7.7%	15.4%	23.1%	7.7%	15.4%
**1 wk (n = 15)**	20.0%	20.0%	13.8%	13.3%	6.7%	13.3%
**2–3 wks (n = 145)**	16.6%	13.1%	11.7%	22.1%	17.2%	17.2%
**4 wks (n = 25)**	12.0%	24.0%	16.0%	24.0%	8.0%	16.0%
**6 wks (n = 33)**	10.7%	32.1%	14.3%	7.1%	10.7%	25.0%
**8 wks (n = 9)**	11.1%	11.1%	22.2%	11.1%	11.1%	22.2%
**12–16 wks (n = 19)**	10.5%	5.3%	10.5%	31.6%	21.1%	10.5%

a—large, identified injured (right) RS neurons (Müller cells; see text and Figure 1B): M cells; I cells; B cells (see Figure 1A); b—percentage of recordings made from a particular injured RS neuron at a particular recovery time (in some cases, the percentages for a given recovery time do not add up to 100% because of a few additional recordings that were made from other smaller Müller cells).
